# Recurrent myositis triggered by infections: a case report

**DOI:** 10.1186/1752-1947-2-344

**Published:** 2008-11-14

**Authors:** Sui H Wong, Bryan RF Lecky, Ian J Hart, Daniel Crooks, Tom Solomon

**Affiliations:** 1 The Walton Centre for Neurology and Neurosurgery NHS Trust, Lower Lane, Fazakerley, Liverpool L7 9LJ, UK; 2 Royal Liverpool University Hospital, Prescot Street, Liverpool L7 8XP, UK

## Abstract

**Introduction:**

Recurrent myositis triggered by infections is unusual, with only one other case reporting two attacks described in the literature.

**Case presentation:**

We report the case of a 24-year-old Caucasian woman with recurrent myositis triggered by sore throat, respiratory and urinary tract infections, over the past 18 years, up to four times a year. Myositis of this frequency and duration, apparently triggered by infections, has not been reported previously.

**Conclusion:**

We believe that this case adds to the understanding of myositis associated with infections being a triggered autoimmune response, and postulate that the pathogenesis in our patient is a non-specific immune response to a range of different precipitants, both bacterial and viral.

## Introduction

Myositis can occur in association with infections. However, its pathogenesis is not clearly understood, and may be due to direct pathogenic invasion, for example, bacterial micro abscesses, or an autoimmune antigenic response. There may be serious consequences including rhabdomyolysis and acute renal failure. Recurrent myositis with different infections may suggest an autoimmune response from antigenic triggers. We describe such a case.

## Case presentation

A 24-year-old woman was referred in 2005 with increasing episodes of debilitating lower limb myalgia, since the age of seven. The attacks occurred every 1–2 years but had increased since 2005 to four per year. They were preceded by sore throats, and more recently, by cough or dysuria. During these episodes, creatine kinase (CK) ranged from 89 to 700 U/litre (normal range <175), normal between episodes. Her muscle pain responded to oral prednisolone within days.

Because of the sore throats and an elevated antistreptolysin O titre (ASOT), she underwent a tonsillectomy in 1992. Apart from the tonsillectomy, her past medical history and drug history were unremarkable.

We set out to fully investigate one of these attacks. In September 2005, an attack of muscle pains was preceded by cough and green sputum 2 weeks earlier. An examination was normal except for a slightly injected throat and tenderness of the calves and anterior compartment muscles. Biopsy of the right tibialis anterior muscle showed mild muscle fibre atrophy of uncertain significance. Erythrocyte sedimentation rate (ESR) was elevated (39 mm/hour), leucocyte count was 16.6 × 10^9^/litre (4.0–11.0), with a neutrophilia of 13.6 × 10^9^/litre (81.9%) and a monocytosis of 1.06 × 10^9^/litre (6.4%). Lymphocyte, eosinophil, basophil counts, CK, renal and liver function were normal.

Serology was negative for influenza A & B, respiratory syncytial virus (RSV), *Mycoplasma pneumoniae*, chlamydia, Q fever, adenovirus, enterovirus, and ASOT.

Acute serology for Epstein-Barr Virus (EBV) was less clear. Epstein-Barr Nuclear Antigen (EBNA) IgG, Viral Capsid Antigen (VCA) IgG and IgM were present. This may be due to a false positive VCA IgM, a primary EBV infection in the last 3–12 months, or a reactivation of EBV. Convalescent EBV serology 5 months later showed a positive EBV VCA and a negative EBV VCA IgM, consistent with past EBV infection.

Due to the uncertain significance of the EBV serology, further investigations were done during an episode in September 2006, triggered after 4 days of urinary tract infection.

On examination, she had a temperature of 38°C. Examination of her throat, skin, abdomen and cardiorespiratory systems was normal. There was marked tenderness of forearm, thighs and calf muscles. Testing lower limb power was inhibited by pain, but was otherwise unremarkable. Tendon reflexes were normal.

Blood tests showed an elevated CK (715 U/litre), leukocytosis of 31.1 × 10^9^/litre (normal range 4–11), neutrophilia (88.6%), lymphocytosis (5.8%) and monocytosis (5.4%). ESR was 80 mm/hour and C-reactive protein was 287 mg/litre. Blood film showed normochromic normocytic red blood cells, leukocytosis with a neutrophilia and monocytosis, occasional atypical mononuclear cells and some rouleaux. Her mid-stream urine (after 7 days of trimethoprim) showed a white cell count of 284 × 10^6^/litre, epithelial cells of 272 × 10^6^/litre and lactobacillus at >100 × 10^6^/litre (probably a contaminant).

Serology for EBV and cytomegalovirus (CMV) confirmed past infection only, with raised IgG and negative IgM. Polymerase chain reaction (PCR) for EBV, adenovirus, and CMV of EDTA plasma was negative. The monospot test for heterophile antibodies (a marker of primary EBV infection) in serum was negative. A throat swab was negative for EBV (by PCR).

**Figure 1:**
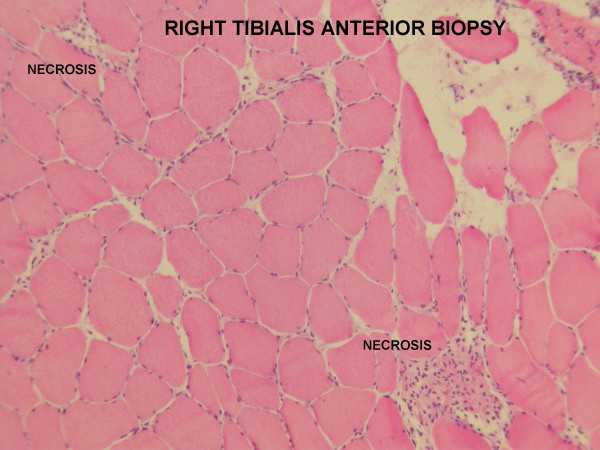
Muscle biopsy of right tibialis anterior (haematoxylin and eosin stain) showing changes consistent with multifocal myonecrosis, of marked interstitial inflammation mainly by CD4 lymphocyte with occasional fibre necrosis associated with CD4 inflammation.

Biopsy of the right tibialis anterior muscle showed marked interstitial inflammation mainly by CD4 lymphocyte with occasional fibre necrosis associated with CD4 inflammation, changes consistent with multifocal myonecrosis (Figure 1). PCR of muscle was negative for EBV and CMV.

Other investigations in between episodes were non-contributory. These included normal CK, full blood count, random blood glucose and autoantibodies. An underlying immunodeficiency was excluded, with normal immunoglobulins, lymphocyte subsets, in vitro lymphocyte proliferation in response to phytohaemagglutinin and pokeweed mitogen. When she was well, electromyography (EMG) of her right tibialis anterior and right medial gastrocnemius muscles in May 2006 showed myopathic changes.

## Discussion

Isolated attacks of myositis associated with infections have been described with most known infection agents [1,2]. However, the pathogenesis and possible underlying trigger mechanisms remain poorly understood. It may be due to direct muscle invasion of the pathogen (for example, foci of myonecrosis and abscesses in bacterial infections), or indirect damage through toxin release or an autoimmune response [1,2]. There may be serious consequences, including rhabdomyolysis and acute renal failure [3]. An autoimmune inflammatory cause may respond to steroids.

However, recurrent myositis associated with infections is rare. The only other case reported was of a patient with two episodes of myositis associated with Mycoplasma and hepatitis A infection [4].

Patients with metabolic myopathies such as fatty acid oxidative disorders may have symptoms triggered by infections or other states of metabolic stress. We felt this was unlikely in our patient as there was no histochemical evidence of any metabolic myopathy.

We postulate that our patient has an autoimmune-mediated myositis triggered by antigenic stimulus from a range of infections (including bacterial and viral triggers).

The initial history of myositis preceded by sore throats prompted us to hunt for EBV as a trigger. Chronic active EBV infections causing polymyositis have been described [5,6]. We later discounted EBV as the sole causative agent, with the absence of EBV in blood, throat and affected muscle and EBV serology showing evidence of past infection. Additionally, our patient later had attacks triggered by urinary tract infections.

There does not appear to be a reason for increased susceptibility to urinary tract infections, with normal cystoscopy and radiological investigations, and she does not have any evidence of immunodeficiency from detailed immunological investigations.

## Conclusion

We describe an unusual case of recurrent myositis triggered by infections. This unusual case further adds to the understanding of myositis associated with infections, and we postulate that the pathogenesis is an over-reactive immune response triggered by a range of antigenic stimuli, both bacterial and viral.

## Abbreviations

ASOT: antistreptolysin O titre; CK: creatine kinase; CMV: cytomegalovirus; EBNA: Epstein-Barr Nuclear Antigen; EBV: Epstein-Barr Virus; EMG: electromyography; ESR: erythrocyte sedimentation rate; PCR: polymerase chain reaction; RSV: respiratory syncytial virus; VCA: Viral Capsid Antigen

## Consent

Written informed consent was obtained from the patient for publication of this case report and any accompanying images. A copy of the written consent is available for review by the Editor-in-Chief of this journal.

## Competing interests

The authors declare that they have no competing interests.

## Authors' contributions

SHW, BRFL, and TS were involved in the clinical management of the patient. IJH analyzed and interpreted the serological and microbiological data. DC performed the histological examination of the muscle biopsy. All authors contributed to the writing of the manuscript.
